# Effect of Different Types of Intermittent Fasting on Biochemical and Anthropometric Parameters among Patients with Metabolic-Associated Fatty Liver Disease (MAFLD)—A Systematic Review

**DOI:** 10.3390/nu14010091

**Published:** 2021-12-26

**Authors:** Gracjan Różański, Derek Pheby, Julia L. Newton, Modra Murovska, Paweł Zalewski, Joanna Słomko

**Affiliations:** 1 Department of Exercise Physiology and Functional Anatomy, Ludwik Rydygier Collegium Medicum in Bydgoszcz Nicolaus Copernicus University in Torun, Świętojańska 20, 85-077 Bydgoszcz, Poland; p.zalewski@cm.umk.pl (P.Z.); jslomko@cm.umk.pl (J.S.); 2 Society and Health, Buckinghamshire New University, High Wycombe HP11 2JZ, UK; derekpheby@btinternet.com; 3 Population Health Sciences Institute, The Medical School, Newcastle University, Newcastle-upon-Tyne NE2 4AX, UK; julia.newton@ahsn-nenc.org.uk; 4 Institute of Microbiology and Virology, Riga Stradiņš University, LV-1067 Riga, Latvia; Modra.Murovska@rsu.lv

**Keywords:** MAFLD, intermittent fasting, diet, liver

## Abstract

Metabolic-associated fatty liver disease (MAFLD), previously called non-alcoholic fatty liver disease (NAFLD), is the most common chronic liver disease worldwide. It is characterised by excessive fat accumulation in hepatocytes. Currently, no pharmacological therapy is effective for this disease, so non-pharmacological alternatives such as diet, supplementation or physical activity are being sought. For this reason, we reviewed the available databases to analyse the studies conducted to date using different modifications of intermittent fasting among patients with MAFLD. Eight studies using this dietary strategy were included in this review. The results obtained in the different trials are varied and do not allow a clear determination of the effect of the different types of intermittent fasting on anthropometric and biochemical parameters among patients with MAFLD. However, this type of diet seems to show some therapeutic potential, but further studies are needed.

## 1. Introduction

Metabolic Associated Fatty Liver Disease (MAFLD), formerly non-alcoholic fatty liver disease (NAFLD), is characterised by excessive fat accumulation in hepatocytes (>5% of liver weight), not caused by viral infection, alcohol consumption or medication [1]. It is the most common chronic liver disease in the world [2,3]. MAFLD is becoming a major public health problem as a significant increase in prevalence has been observed in recent years, with annual costs estimated at €35 billion in Europe and €89 billion in the US. These putative costs were determined on the basis of the construction of a Markov model that simulated progression from the healthy to the diseased population, taking into account age-related incidence and progression data, though limitations of the method may result in an underestimation of the true burden [4].

In 2013, it was reported that up to 50% of obese men may suffer from MAFLD [5]. It is also estimated that 23–44% of patients with MAFLD will develop non-alcoholic steatohepatitis (NASH), subsequently leading to fibrosis and, in the worst case, cirrhosis, which will lead to liver failure within 5–7 years in 40–60% of individuals and, within 3–7 years, in 2.4–12% of patients, to hepatocellular carcinoma (HCC) [6]. The European Association for the Study of the Liver recommends dietary changes and a progressive increase in aerobic exercise or resistance training in patients with MAFLD [7]. Factors such as early diagnosis, prevention, the treatment of risk factors and lifestyle changes are also important [4], especially because of the association of MAFLD with, among others, insulin resistance, dyslipidaemia, diabetes, hypertension or metabolic syndrome [8,9,10,11,12] and increased risk of liver- and cardiovascular disease-related mortality [13,14].

### 1.1. Pathophysiology: Multiple Hits Hypothesis

The incompletely understood pathogenesis of MAFLD, due to its complexity and the involvement of multiple factors, is referred to as the “multiple hits hypothesis”. Dietary habits and genetic and environmental factors may influence the development of the disease, but insulin resistance (IR) is currently identified as the main cause. It leads to greater de novo hepatic lipogenesis (DNL) and a weaker inhibition of adipose tissue lipolysis, resulting in an increased flow of fatty acids to the liver and an accumulation of triglycerides in hepatocytes. Furthermore, IR leads to the altered production and secretion of adipokines and pro-inflammatory cytokines. The lipotoxicity resulting from high levels of free fatty acids, free cholesterol and other lipid metabolites is increased, leading to the production of reactive oxygen species that are responsible for mitochondrial and endoplasmic reticulum dysfunction. The intestinal microbiota is also not insignificant, as it has been shown that changes within it lead to an increase in the permeability of the small intestine and the resulting increased absorption of fatty acids and higher levels of circulating molecules. This results in the activation of inflammatory pathways and the release of i.a. IL-6 and TNF-α [12].

### 1.2. Intermittent Fasting

Intermittent fasting is a feeding method that uses periods of reduced energy supply from food. It can use various forms of food restriction. One of them is alternate-day fasting (ADF), in which fasting is applied every other day. During the “fasting days”, energy-providing products should not be consumed, and during the “feeding days”, food may be eaten ad libitum. A kind of ADF variance is Alternate-Day Modified Fasting (ADMF), which on fasting days allows energy intake at the level of 25–30% in the “open” eating window, usually for 2–4 h. Another method of intermittent fasting is modified fasting regimens (MFR), which enables energy consumption to cover 20–25% of the daily energy requirement during regularly scheduled fasting days. This method is used in a 5:2 diet, restricting food intake for 2 days a week and with usual eating for the remaining 5 days. Another strategy is time-restricted feeding (TRF), which sets specific time frames for eating and fasting, e.g., 8 h and 16 h, respectively, with no energy restriction. The methods of fasting also include religious fasting, such as Ramadan, whose rules allow eating only after sunset, but before dawn. It is a kind of time-restricted feeding, because fasting and eating periods last about 12 h and the amount of energy supplied is not limited [15,16,17].

## 2. Materials and Methods

### 2.1. Types of Participants

Studies were included if they were conducted in adult patients (aged >18) of any gender or nationality with metabolic-associated fatty liver disease (MAFLD).

### 2.2. Types of Interventions

Interventions using any kind of intermittent fasting and presenting the results of biochemical parameters or anthropometric measurements at baseline and after fasting were included. Animal trials and studies that recommended other changes such as lifestyle changes or physical activity were excluded.

### 2.3. Types of Comparisons

No specific comparison criteria.

### 2.4. Types of Outcomes

The outcomes of at least one biochemical parameter or anthropometric measurements were presented in the study, measured at baseline (pre-fasting period) and after a post-fasting period.

### 2.5. Types of Studies

Any type of study (apart from case reports and reviews) was included if it was a study published in a peer-reviewed journal in English. There were no restrictions on intervention length or follow-up measurement points. The exclusion criteria were as follows: non-human studies, the use of an additional intervention other than any kind of intermittent fasting, such as lifestyle changes or physical activity. The PICOS criteria for inclusion and exclusion of studies are shown in Table 1.

### 2.6. Search Strategy and Study Selection

**Figure 1:**
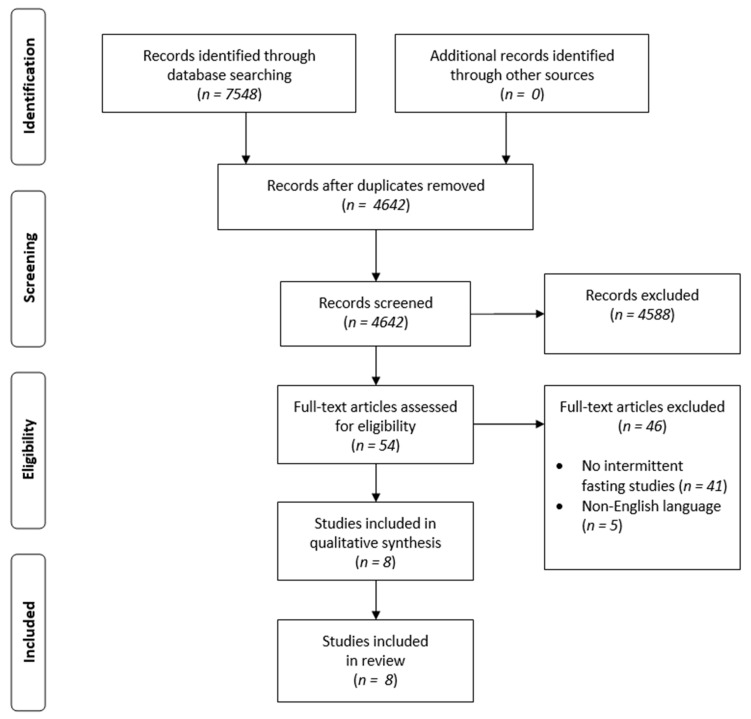
PRISMA flow diagram of the study selection.

We reviewed available publications using the databases PubMed, Web of Science and Scopus, using the words “NAFLD”, “MAFLD”, “metabolic-associated fatty liver disease” or “non-alcoholic fatty liver disease”, and “intermittent fasting”, “Ramadan” or “time-restricted eating”. We limited the results to papers in English (Figure 1). Flow diagrams for each database are available in Supplementary Materials.

## 3. Results

### 3.1. Study Selection

Eight studies that used intermittent fasting in patients with MAFLD were included in this review. In the majority of the papers studied, the fasting regimen reported was Ramadan. Other regimens reported were ADMF (Alternate-Day Modified Fasting), TRF (time-restricted fasting) and MFR (modified fasting regimen).

### 3.2. Participant Characteristics

In two studies, the mean age of patients ranged from 33.6–37.6 years [18,19]. In another four studies, it averaged 40.5–46.0 years [20,21,22,23] and in two studies the age range was 51.8–57.0 years [24,25]. The mean BMI of study participants ranged from 29.5–36.7 kg/m^2^. Information on the study groups is provided in Table 2.

### 3.3. Fasting Strategy and Duration

Five studies used Ramadan as an intervention, which lasted 4 weeks [19,20,21,22,25]. One study (in one group) used TRF lasting 12 weeks [18]. Two studies used ADMF: Cai et al. (in the second group) lasting 12 weeks [18] and Johari et al. lasting 8 weeks [23]. Additionally, Holmer et al. used MFR for 12 weeks [24]. Detailed information on the included studies can be found in Table 3.

### 3.4. Time-Restricted Fasting (TRF)

Time-restricted fasting was used in six studies [18,19,20,21,22,25]. Five of them used Ramadan as a form of intermittent fasting [19,20,21,22,25] which lasted about 4 weeks. The study by Cai et al. [18] lasted 12 weeks, but patients were also followed up after 4 weeks of TRF.

Body weight was controlled in three studies [18,19,20]. Arabi et al. in their two studies [21,22] and Mari et al. [25] reported only the BMI value. Aliasghari et al. [19] reported a statistically significant decrease in the mean body weight of patients after 4 weeks of Ramadan (83.7 ± 13.0 kg vs. 81.5 ± 12.8 kg, *p* < 0.001), with a statistically significant decrease in BMI (30.1 ± 4.5 kg/m^2^ vs. 29.3 ± 4.1 kg/m^2^, *p* < 0.001). Cai et al. [18] also observed a statistically significant decrease in body weight compared to the control group after both 4 weeks (75.0 ± 8.0 kg vs. 71.3 ± 7.0 kg, *p* < 0.001) and 12 weeks (75.0 ± 8.0 kg vs. 71.7 ± 7.4 kg, *p* < 0.001). Rahimi et al. [20] reported no statistically significant change in body weight in the study group compared to the control group. Arabi et al., in both studies, [21,22] reported no statistically significant change in BMI values, in contrast to Mari et al. [25], who reported a statistically significant decrease (36.7 ± 7.1 kg/m^2^ vs. 34.5 ± 6.8 kg/m^2^, *p* < 0.005).

Waist circumference was monitored in three studies [18,19,22]. Only Aliasghari et al. [19] obtained a statistically significant change (100.2 ± 11.0 cm vs. 99.3 ± 10.9 cm, *p* < 0.001). The other studies also observed a reduction in waist circumference, but the results were not statistically significant. Furthermore, Aliasghari et al. [19] also controlled for hip circumference, observing a statistically significant decrease (107.0 ± 7.1 cm vs. 106.5 ± 7.1 cm, *p* < 0.001). They also calculated the waist:hip ratio, also noting a statistically significant decrease (0.9 ± 0.1 vs. 0.9 ± 0.1, *p* < 0.001).

Cai et al. [18] measured fat mass, in which they reported a statistically significant decrease after 4 weeks (30.3 ± 3.2 kg vs. 27.4 ± 3.4 kg, *p* < 0.001) and 12 weeks (30.3 ± 3.2 kg vs. 27.7 ± 3.3, *p* < 0.001). Arabi et al. in one study [22] and Aliasghari et al. [19] measured body fat content. Arabi et al. [22] found no statistically significant change in both men (24.2% vs. 24.2%, *p* = 0.63) and women (41.7% vs. 41.1%, *p* = 0.23). However, Aliasghari et al. [19] reported statistically significant differences: in men (29.8 ± 6.6% vs. 29.0 ± 6.6%, *p* < 0.001) and women (41.9 ± 6.2% vs. 41.4 ± 6.1%, *p* = 0.03). Arabi et al. [22] also found no statistically significant change in free fatty mass (men: 65.2 ± 9.6% vs. 64.5 ± 10.0%, *p* = 0.10; women: 48.0 ± 6.7% vs. 49.4± 6.33%, *p* = 0.12). Cai et al. [18] also reported no statistically significant changes in lean mass.

Arabi et al. [22] monitored systolic blood pressure (SBP) and diastolic blood pressure (DBP) levels in their study. They noted significant differences only in the male group. SBP at baseline was 130 mmHg, and after intervention it was 120 mmHg (*p* < 0.001). DBP decreased from 70.50 mmHg at baseline to 70.00 mmHg (*p* = 0.003). In women, SBP was at baseline and after intervention 120 mmHg, while DBP increased from an initial 78 mmHg to 80 mmHg (*p* = 0.75).

ALT levels were determined by Rahimi et al. [20], Arabi et al. [22] and Mari et al. [25]. Statistically significant differences were noted in all studies. In the first study, there was an increase in ALT level (34.6 ± 13.5 IU/L vs. 42.0 ± 17.8 IU/L; change = 7.4 ± 8.5 IU/L—statistically significant compared to the change in the control group; *p* = 0.002), while decreases were observed in the second and third studies. Arabi et al.: in men (18 IU/L vs. 13 IU/L, *p* < 0.001), in women (13 IU/L vs. 11 IU/L, *p* = 0.001) [22]; Mari et al.: 44.2 ± 12.8 IU/L vs. 34.2 ± 9.4 IU/L, *p* < 0.005 [25]. Arabi et al. [22] also measured AST levels but did not find a statistically significant change in either group, whereas Mari et al. found a statistically significant decrease (51.4 ± 9.4 IU/L vs. 39.2 ± 8.2 IU/L, *p* < 0.005) [25].

Fasting blood glucose levels were monitored in three studies [18,19,22]. Cai et al. [18] reported no statistically significant change, while Arabi et al. [22] and Aliasghari et al. [19] observed statistically significant increases (85.5 mg/dL vs. 133.6 mg/dL, *p* < 0.001 in men; 100 mg/dL vs. 120.2 mg/dL, *p* < 0.001 in women; and 94.0 ± 8.0 mg/dL vs. 92.0 ± 7.8 mg/dL, *p* < 0.001, respectively). Aliasghari et al. [19], Arabi et al. [22] and Mari et al. [25] also measured insulin levels and noted changes, which in the latter two cases were statistically significant decreases: Aliasghari et al.: 15.1 ± 2.8 IU/mL vs. 15.3 ± 2.8 IU/mL, *p* < 0.001; Arabi et al. in the female group: 15.9 ± 7.1 mg/dL vs. 12.7 ± 4.6 mg/dL, *p* = 0.01 and Mari et al.: 24.7 ± 5.2 IU/mL vs. 20.3 ± 3.3 IU/mL, *p* < 0.005. In addition, Aliasghari et al. [19] and Mari et al. [25] also controlled for HOMA-IR and noted small decreases which were not statistically significant: Aliasghari et al.: 3.5 ± 0.7 vs. 3.5 ± 0.7, *p* = 0.011 and Mari et al.: 2.9 ± 1.2 vs. 2.2 ± 1.1, *p* < 0.005.

Cai et al. [18] and Arabi et al. [22] measured triglyceride levels. Both studies reported differences after the intervention, but Cai et al. reported a statistically significant decrease compared to the control group at both 4 and 12 weeks (baseline: 2.9 ± 1.8 mmol/L; after 4 weeks: 2.3 ± 1.8 mmol/L, *p* < 0.001; after 12 weeks: 2.3 ± 1.8 mmol/L, *p* < 0.001) [18], while Arabi et al. reported an increase in triglyceride levels (138 mg/dL vs. 190 mg/dL, *p* < 0.001 in men and 197 mg/dL vs. 233 mg/dL, *p* = 0.23 in women) [22].

Total cholesterol levels were again only measured by Cai et al. [18] and Arabi et al. [22]. The first study reported no statistically significant change compared to the control group [18], whereas Arabi et al. observed statistically significant increases in levels in both men and women (190.6 ± 47.4 mg/dL vs. 220.7 ± 60.0 mg/dL, *p* = 0.001 and 200.0 ± 45.5 mg/dL vs. 229.1 ± 47.9 mg/dL, *p* = 0.001, respectively) [22].

LDL-C and HDL-C concentrations were only measured by Cai et al. [18] and Arabi et al. [22]. The authors of the first study found no statistically significant change in the outcome of the intervention compared to the control group [18]. In contrast, Arabi et al. reported a statistically significant increase in HDL-C levels in women (46.52 ± 10.16 mg/dL vs. 53.9 ± 16.5 mg/dL, *p* = 0.04). The other results were not statistically significant: 42.7 ± 8.1 mg/dL vs. 45.3 ± 10.0 mg/dL, *p* = 0.22 (HDL-C in men), 119.1 ± 33.8 mg/dL vs. 121.6 ± 36.5 mg/dL, *p* = 0.71 (LDL-C in men) and 124.1 ± 23.6 mg/dL vs. 127.7 ± 38.2 mg/dL, *p* = 0.61 (LDL-C in women) [22].

Aliasghari et al. [19] monitored IL-6 and hs-CRP levels in their study, while Mari et al. [25] only measured hs-CRP levels. Aliasghari et al. reported statistically significant decreases in both parameters mentioned (IL-6: 75.4 ± 89.1 ng/mL vs. 74.8 ± 89.1, *p* < 0.001; hs-CRP: 1.6 ± 0.6 mg/L vs. 1.2 ± 0.7 mg/L, *p* < 0.001) [19], Mari et al. also reported a statistically significant decrease in hs-CRP level (14.2 ± 7.1 mg % vs. 7.2 ± 6.5 mg %, *p* < 0.005) [25].

Arabi et al. in their second study [21] measured the levels of palmitic acid, oleic acid and elaidic acid. The intervention reported only one significant statistical change: an increase in elaidic acid levels among women (24.8 ± 2.1 ppm vs. 35.9 ± 9.8 ppm, *p* = 0.001).

### 3.5. Alternate-Day Modified Fasting (ADMF)

Two trials used alternate-day modified fasting (ADMF) as an intervention [18,23]. Cai et al. [18] conducted a trial lasting 12 weeks with a checkpoint after 4 weeks. In contrast, the trial by Johari et al. lasted 8 weeks [23].

Both Johari et al. [23] and Cai et al. [18] reported statistically significant decreases (Cai et al. versus control group) in body weight. Johari et al. reported an initial mean body weight in the study group of 80.8 kg, while after 8 weeks it was 78.8 kg (*p* = 0.003) [23]. Cai et al. reported the following values: Baseline: 75.3 ± 8.5 kg; after 4 weeks: 70.8 ± 7.8 kg, *p* < 0.001; after 12 weeks: 71.3 ± 7.0 kg, *p* < 0.001. For BMI, only Johari et al. [23] obtained a statistically significant change (31.7 vs. 31.0, *p* = 0.003). Cai et al. [18] also measured waist circumference, lean mass and fat mass. For waist circumference and lean mass, they obtained no statistically significant change compared to the control group, but they recorded a statistically significant decrease in fat mass compared to the control group (Baseline: 30.6 ± 4.0 kg; after 4 weeks: 27.1 ± 2.5 kg, *p* < 0.001; after 12 weeks: 27.1 ± 2.5, *p* < 0.001).

ALT and AST levels were only measured by Johari et al. [23]. They obtained statistically significant decreases in both (ALT: 84.3 IU/L vs. 59.2 IU/L, *p* = 0.001; AST: 51.4 IU/L vs. 42.8 IU/L, *p* = 0.004).

In contrast, fasting glucose levels were monitored in both studies [18,23]. Only Johari et al. [23] obtained a statistically significant change (6.6 mmol/L vs. 5.9 mmol/L, *p* = 0.006).

TG, TC, LDL-C and HDL-C were measured in both trials [18,23]. Only Cai et al. [18] reported statistically significant changes in TG in comparison with the control group (Baseline: 2.8 ± 1.9 mmol/L; after 4 weeks: 2.2 ± 1.9 mmol/L, *p* < 0.001; after 12 weeks: 2.1 ± 1.9 mmol/L, *p* < 0.001) and in TC (Baseline: 4.9 ± 1.0 mmol/L; after 4 weeks: 4.0 ± 1.1 mmol/L, *p* < 0.001; after 12 weeks: 4.2 ± 1.1 mmol/L, *p* < 0.001). Otherwise, no statistically significant changes were observed in either sample [18,23].

### 3.6. Modified Fasting Regimens (MFR)

Modified fasting regimens were used in only one trial [24]. The intervention conducted by Holmer et al. used a 5:2 diet for 12 weeks.

The study authors observed statistically significant changes in anthropometric parameters (body weight: 96.9 ± 14.3 kg, change: −7.4 kg (95% CI: −8.7, −6.0), *p* < 0.001; BMI: 32.3 ± 2.7 kg/m^2^, change: −2.4 kg/m^2^ (95% CI: −2.8, −2.0), *p* < 0.001 and waist:hip ratio: 1.0 ± 0.1, change: −0.03 (95% CI: −0.04, −0.01), *p* < 0.001).

No statistically significant changes were reported for SBP and DBP. However, a statistically significant decrease in ALT levels was reported (59 ± 23 IU/L, change: −17.6 IU/L (−29.4, −11.8), *p* < 0.001). AST levels did not change statistically significantly.

After the intervention, a statistically significant change was noted for HOMA-IR (6.8 ± 2.7; change: −3.2 (95% CI: −4.1, −2.2), *p* < 0.001) and HbA1c (42.6 ± 8.9 mmol/mol, change: −4.8 mmol/mol (95% CI: −6.5, −3.0), *p* < 0.001).

The authors also monitored TG, TC, LDL-C and HDL-C levels. Significant changes were observed for TG (1.9 ± 0.6 mmol/L, change: −0.4 mmol/L (95% CI: −0.6, −0.1), *p* = 0.004), TC (5.3 ± 1.2 mmol/L, change: −0.50 mmol/L (95% CI: −0.8, −0.3), *p* < 0.001) and LDL-C (3.2 ± 1.1 mmol/L, change: −0.40 mmol/L (−0.6, −0.2), *p* < 0.001).

## 4. Discussion

To the best of our knowledge, this is the first review of the effect of various modifications of intermittent fasting on biochemical and anthropometric parameters in MAFLD, focusing on dietary-only interventions.

The studies conducted so far determining the influence of nutrition on the development of MAFLD and with the use of various nutritional modifications in the course of this disease indicate that diet is one of the key factors involved in both the development of the disease and its therapy [26,27,28,29]. Therefore, the appropriate composition of the diet is being sought and attempts are being made to determine the effect of individual dietary components in relation to MAFLD. Previous studies have suggested a beneficial effect of consuming mono-unsaturated fatty acid (MUFA) and polyunsaturated fatty acid (PUFA) instead of saturated fatty acids. Another component with a positive effect is dietary fibre, as its low intake is associated with disease progression. For this reason, the consumption of whole grain products, among others, is recommended [30]. There is also no doubt that fructose intake leads to the development and progression of MAFLD; therefore, its daily intake should be controlled in both patients and healthy individuals for prophylactic purposes [27,31]. Patients with MAFLD are also advised to regularly consume fruit and vegetables (with a predominance of vegetables), due to their content of vitamins, minerals, polyphenols and fibre [27,30,31]. In addition to providing nutrients in adequate amounts, an adequate diet should also be balanced in terms of energy, as it should lead to weight reduction in MAFLD patients who are overweight or obese [27].

Intermittent fasting has so far been used in studies in the course of many diseases, e.g., obesity, type 2 diabetes or cardiovascular diseases, with measurable effects [15,16,17,32]. It should be noted that these are diseases that often coexist with MAFLD [33], making it reasonable to examine the use intermittent fasting among patients with MAFLD.

Overweight or obesity is often observed among people with MAFLD, and diet should lead to effective weight reduction [24,33]. Aliasghari et al. (4 weeks of Ramadan) [19] and Cai et al. (12 weeks of TRF in one group and 12 weeks of ADMF in the other group) [18] reported statistically significant (Cai et al. versus control group) decreases in weight in subjects. Mari et al. (4 weeks of Ramadan) reported a statistically significant decrease in BMI. Rahimi et al. (4 weeks of Ramadan) [20] also observed a decrease in weight in patients, although not statistically significant. A meta-analysis including 35 studies that analysed the effect of Ramadan on weight loss in healthy adults found that there was a significant reduction in weight (−1.51 kg for men and −0.92 kg for women). However, this effect did not last longer than 2 weeks after the end of Ramadan, returning to the weight values before the start of this form of intermittent fasting. It was also pointed out that consistent lifestyle changes are necessary to maintain a consistent effect [34]. Cai et al. [18] and Johari et al. [23], using alternate-day modified fasting (ADMF) for 12 and 4 weeks, respectively, reported statistically significant weight decreases among patients in their trials. Cai et al. [18], although they did not obtain statistically significant changes in waist circumference and lean mass, reported statistically significant decreases in fat mass. Parvaresh et al. [35], in their study among individuals with metabolic syndrome, demonstrated that ADMF may be more effective in controlling body weight, waist circumference, systolic blood pressure and fasting plasma glucose, compared with common calorie restriction. In another study, by Razavi et al., also conducted in patients with metabolic syndrome, found that ADMF was more effective in reducing body weight, fat mass, waist circumference and waist:hip ratio compared with common calorie restriction [36]. A meta-analysis by Park et al. including studies using ADM or ADMF showed that this type of intermittent fasting was effective in reducing body weight, BMI and fat mass over 6 months in overweight individuals [37].

Inflammation has been shown to be involved in the development, course and progression of MAFLD. Therefore, an important element of the therapy is its reduction [12]. Razavi et al. also reported ADMF to be more effective in reducing hs-CRP levels [36]. Aliasghari et al. (4 weeks of Ramadan) [19] and Mari et al. (4 weeks of Ramadan) [25] also reported statistically significant decreases in hs-CRP levels in their study. In addition, Aliasghari et al. [19] reported statistically significant decreases in IL-6 levels. A study of 50 healthy individuals adhering to Ramadan also found that levels of the proinflammatory cytokines IL-1β, IL-6 and tumour necrosis factor α were lower during Ramadan [38].

A characteristic of MAFLD is the coexistence of other diseases for which proper nutrition may be helpful. One of them is hypertension [12]. Arabi et al. (4 weeks of Ramadan) [22] controlled for SBP and DBP in patients, noting a significant decrease only for SBP in men. Holmer et al. (MFR) [24] reported no statistically significant changes in SBP and DBP. However, a meta-analysis by Kord-Varkaneh et al. found that the use of fasting and energy restricting diets significantly reduces SBP and DBP, noting that interventions lasting ≤12 weeks were more effective [39]. In their meta-analysis, Moon et al. also noted that TRF reduced SBP [40].

ALT and AST are considered to be among the most important markers of liver function. For this reason, the effectiveness of therapeutic management is often assessed by the effect on these two parameters. Among studies controlling for the effect of Ramadan on patients, some differences were observed for ALT. Arabi et al. [22] and Mari et al. [25] reported statistically significant decreases, while Rahimi et al. [20] reported a statistically significant increase in ALT levels compared to the change in the control group. Johari et al. [23], using ADMF in their intervention, observed a statistically significant decrease in ALT levels. The same effect was obtained by Holmer et al. [24] studying the effect of MFR. For AST, Arabi et al. (4 weeks of Ramadan) [22] observed no statistically significant change. This is in contrast to Mari et al. (4 weeks of Ramadan) [25], who reported a statistically significant decrease in AST levels. Johari et al. (ADMF) [23] also observed a statistically significant decrease in AST levels. A study by Holmer et al. (MFR) [24] found no statistically significant change in AST levels. A meta-analysis of 20 studies among healthy individuals doing Ramadan found that this form of intermittent fasting leads to significant but small positive changes in ALT and AST [41].

As mentioned earlier, other comorbidities are also observed in the course of MAFLD. Apart from obesity or hypertension, dysplimidaemia can also be observed among patients [12]. For TG, differences were observed between the studies of Cai et al. (TRF) [18] and Arabi et al. (4 weeks of Ramadan) [22]. The authors of the first study reported a statistically significant decrease in TG levels [18] while the second study reported an increase [22]. Cai et al. [18] also observed a statistically significant decrease in TG levels among patients using ADMF. Johari et al. (ADMF) [23] reported no statistically significant change. Holmer et al. (MFR) [24] also reported a statistically significant decrease in TG levels.

Cai et al. (TRF) [18] reported no statistically significant change in TC levels, while Arabi et al. (4 weeks of Ramadan) observed statistically significant increases in TC levels in women and men. Cai et al. [18], in their second group of patients (ADMF), reported a statistically significant decrease in TC levels, which was not reported by Johari et al. [23]. Holmer et al. (MFR) [24] observed a statistically significant decrease in TC levels.

LDL-C and HDL-C was controlled by Cai et al. (TRF and ADMF) [18], Arabi et al. (4 weeks Ramadan) [22], Johari et al. (ADMF) [23] and Holmer et al. (MFR) [24]. For studies using TRF, the only statistically significant change was reported by Arabi et al.: there was an increase in HDL-C levels in the female group [22]. No statistically significant changes in LDL-C and HDL-C levels were reported after ADMF [18,23]. Holmer et al. (MFR) [24] observed a statistically significant decrease in LDL-C.

A meta-analysis of studies that used Ramadan showed that it may cause moderate improvements in lipid and lipoprotein parameters, especially HDL-C levels. Furthermore, according to the authors, Ramadan appears to be more effective in male and athletic subjects [42]. In their meta-analysis of studies using ADF or ADMF, Park et al. also showed that these methods are effective in lowering TC levels within 6 months in overweight individuals [37]. A meta-analysis by Moon et al. showed that TRF resulted in lower TG levels but had no effect on LDL-C and HDL-C [40].

An important factor, even considered as the basis of MAFLD pathogenesis, is insulin resistance. Therefore, one of the therapeutic goals should be to increase the insulin sensitivity of tissues and to aim for normal fasting glucose values [12]. Statistically significant increases in fasting blood glucose levels were reported by Arabi et al. (4 weeks of Ramadan) [22] and Aliasghari et al. (4 weeks of Ramadan) [19]. Cai et al. [18] in both groups (TRF and ADMF) reported no statistically significant changes. In contrast to Cai et al., Johari et al. (ADMF) [23] obtained a statistically significant decrease in fasting blood glucose levels. Furthermore, Holmer et al. (MFR) [24] obtained a statistically significant decrease in HbA1c levels.

For insulin levels, Aliasghari et al. (4 weeks of Ramadan) [19] reported a statistically significant increase, in contrast to Arabi et al. (4 weeks Ramadan) [22], who obtained a statistically significant decrease in the female group and Mari et al. (4 weeks of Ramadan) [25], who also found a decrease.

Aliasghari et al. (4 weeks of Ramadan) [19], Mari et al. (4 weeks of Ramadan) [25] and Holmer et al. (MFR) [24] also reported statistically significant decreases in HOMA-IR.

Moon et al. [40], in a meta-analysis, showed that TRF has a positive effect on fasting blood glucose. A meta-analysis by Faris et al. [43] showed that Ramadan observance can positively affect glucose and insulin levels and HOMA-IR. A meta-analysis of studies among individuals without chronic metabolic disease also found that intermittent fasting significantly improves glycemic control and insulin resistance [44]. Another meta-analysis showed that intermittent fasting may improve glycemic control among obese individuals with type two diabetes. However, it was noted that this dietary strategy has similar effects to simple energy restriction and may not be effective in the long term [45].

Intermittent fasting is a quite specific diet, which due to its principles (e.g., long periods of fasting), may not suit all patients; therefore, the validity of its use should be confirmed by research. On the basis of the studies conducted so far on the effect of intermittent fasting in the course of metabolic diseases, it is difficult to unequivocally determine the effects of this dietary strategy. The results so far indicate that intermittent fasting may have positive effects, but this is not the rule. It is also uncertain whether intermittent fasting produces more positive results compared to other diets. At present, intermittent fasting cannot certainly be recommended as an effective method of improving a patient’s condition, regardless of the diseases represented by the patient, but this diet cannot be categorically rejected either.

## 5. Study Limitations

Our study has several strengths. First of all, the effects of specific types of intermittent fasting on biochemical and anthropometric parameters were analysed; intermittent fasting in general was not analysed. Only studies involving humans were included in the study. All studies included in this review used only intermittent fasting as an intervention. A limitation of this review is the small number of studies overall and for specific types of intermittent fasting and the presence of differences between study groups (age, BMI).

## 6. Conclusions

Intermittent fasting appears to show some therapeutic potential among patients with MAFLD. However, at the moment it is not possible to determine unequivocally the effect of this dietary strategy on biochemical and anthropometric parameters, as the results of studies conducted to date are inconsistent, and there is only a very small number of relevant published reports of randomised controlled trials. The effects of modifications of intermittent fasting should clearly be given separate consideration, and not simply lumped together under the overall heading of intermittent fasting. Furthermore, it is not yet possible to determine the effect of intermittent fasting in the long term. Therefore, further studies on numerous groups of patients with longer-term use of the intervention are needed.

## Figures and Tables

**Table 1:** PICOS criteria for inclusion and exclusion of studies.

Parameter	Defined Criteria for the Current Study
P (population)	Adult patients with MAFLD
I (intervention	Any type of intermittent fasting
C (comparison)	No special comparison criteria
O (outcomes)	Changes in biochemical parameters or anthropometric measurements *
S (study design)	Any type apart from case reports and reviews

* ALT, alanine aminotransferase; AST, aspartate aminotransferase; BMI, Body Mass Index; CT, clinical trial; DBP, diastolic blood pressure; FBG, fasting blood glucose; HbA1c, glycated hemoglobin; HDL-C, high-density lipoprotein cholesterol; hs-CRP, high-sensitivity C-reactive protein; LDL-C, low-density lipoprotein cholesterol; RCT, randomised controlled trial; SBP, systolic blood pressure.

**Table 2:** Characteristic of studied groups.

	*n*	Age (year)	BMI (kg/m^2^)
Rahimi, 2017 [20]	34 (25 men, 9 women)	46.03 ± 11.72	29.46 ± 4.52
Cai, 2019; ADMF group [18]	95 (35 men, 60 women)	35.50 ± 4.417	26.12 ± 2.21
Cai, 2019; TRF group [18]	95 (29 men, 66 women)	33.56 ± 6.23	26.76 ± 1.59
Arabi, 2016 [21]	50 (33 men, 17 women)	40.52 ± 10.90	31.38 ± 4.9
Arabi, 2016 [22]	50 (33 men, 17 women)	40.52 ± 10.90	29.50 (men) 34.15 (women)
Aliasghari, 2017 [19]	42 (25 men, 17 women)	37.59 ± 7.06	30.09 ± 4.49
Johari, 2019 [23]	33 (24 men, 9 women) *	45.33 ± 10.77	31.73
Holmer, 2021 [24]	25 (13 men, 12 women)	57 ± 10	32.3 ± 2.7
Mari, 2021 [25]	74 (39 men, 35 women)	51.8 ± 20.9	36.7 ± 7.1

* 3 drop-outs before final analysis.

**Table 3:** Characteristics of included studies.

	Study Design	Type of Intervention	Duration	*n* (Study Group)	*n* (Control Group)	Tested Parameters
Rahimi, 2017 [20]	Prospective observational cross-sectional study	Ramadan	4 weeks	34	26	Body weight, BMI, ALT
Cai, 2019 [18]	RCT	ADMF	12 weeks	95	79	Body weight, BMI, WC, Fat mass, Lean mass, FBG, TG, TC, LDL, HDL
		TRF		95	79	
Arabi, 2016 [21]	Cross-sectional study	Ramadan	27.3 ± 5 days (25–30)	50	-	BMI, Palmitic acid, Oleic acid, Elaidic acid
Arabi, 2016 [22]	Prospective observational cross-sectional study	Ramadan	27.3 ± 5 days (25–30)	50	-	BMI, WC, Fat mass, Free fatty mass, SBP, DBP, ALT, AST, FBG, Insulin, TG, HDL, LDL
Aliasghari, 2017 [19]	Observational trial	Ramadan	4 weeks	42	41	Body weight, BMI, WC, Waist:Hip ratio, Fat mass, FBG, Insulin, HOMA-IR, IL-6, hs-CRP
Johari, 2019 [23]	RCT	ADMF	8 weeks	33 *	10	Body weight, BMI, ALT, AST, FBG, TG, TC, LDL, HDL
Holmer, 2021 [24]	RCT	MFR	12 weeks	25	24	Body weight, BMI, Waist:hip ratio, SBP, DBP, ALT, AST, HOMA-IR, HbA1c, TG, TC, LDL-C, HDL-C
Mari, 2021 [25]	Retrospective, case-control study	Ramadan	4 weeks	74	81	BMI, ALT, AST, Insulin, HOMA-IR, hs-CRP

* 3 drop-outs. ALT, alanine aminotransferase; AST, aspartate aminotransferase; BMI, Body Mass Index; CT, clinical trial; DBP, diastolic blood pressure; FBG, fasting blood glucose; HbA1c, glycated hemoglobin; HDL-C, high-density lipoprotein cholesterol; hs-CRP, high-sensitivity C-reactive protein; LDL-C, low-density lipoprotein cholesterol; RCT, randomised controlled trial; SBP, systolic blood pressure; TC, total cholesterol; TG, total triglycerides; WC, waist circumference.

## Supplementary Materials

The following are available online at www.mdpi.com/article/10.3390/nu14010091/s1, Figure S1: PRISMA flow diagram of the study selection from PubMed database; Figure S2: PRISMA flow diagram of the study selection from Scopus database; Figure S3: PRISMA flow diagram of the study selection from Web of Science database.
