# Supplementary material for: Effect of Different Types of Intermittent Fasting on Biochemical and Anthropometric Parameters among Patients with Metabolic-Associated Fatty Liver Disease (MAFLD)—A Systematic Review
Source: Nutrients. 2021 Dec 26;14(1):91. doi: 10.3390/nu14010091 (PMC8747070; doi:10.3390/nu14010091)
Supplement: Supplementary file 1 [file nutrients-14-00091-s001.zip › nutrients-1503774-supplementary.pdf]

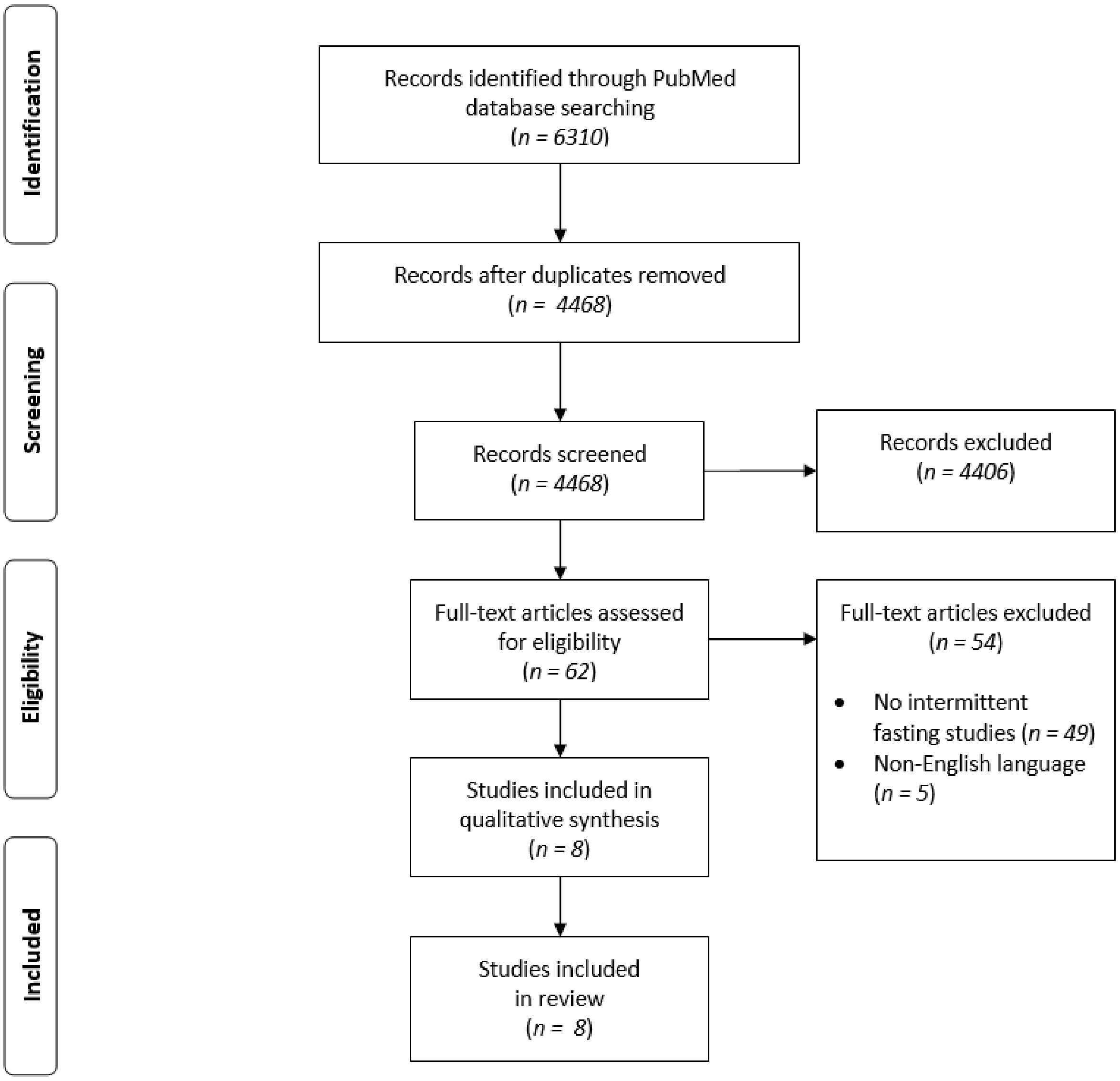

Figure S1. PRISMA flow diagram of the study selection from PubMed database.

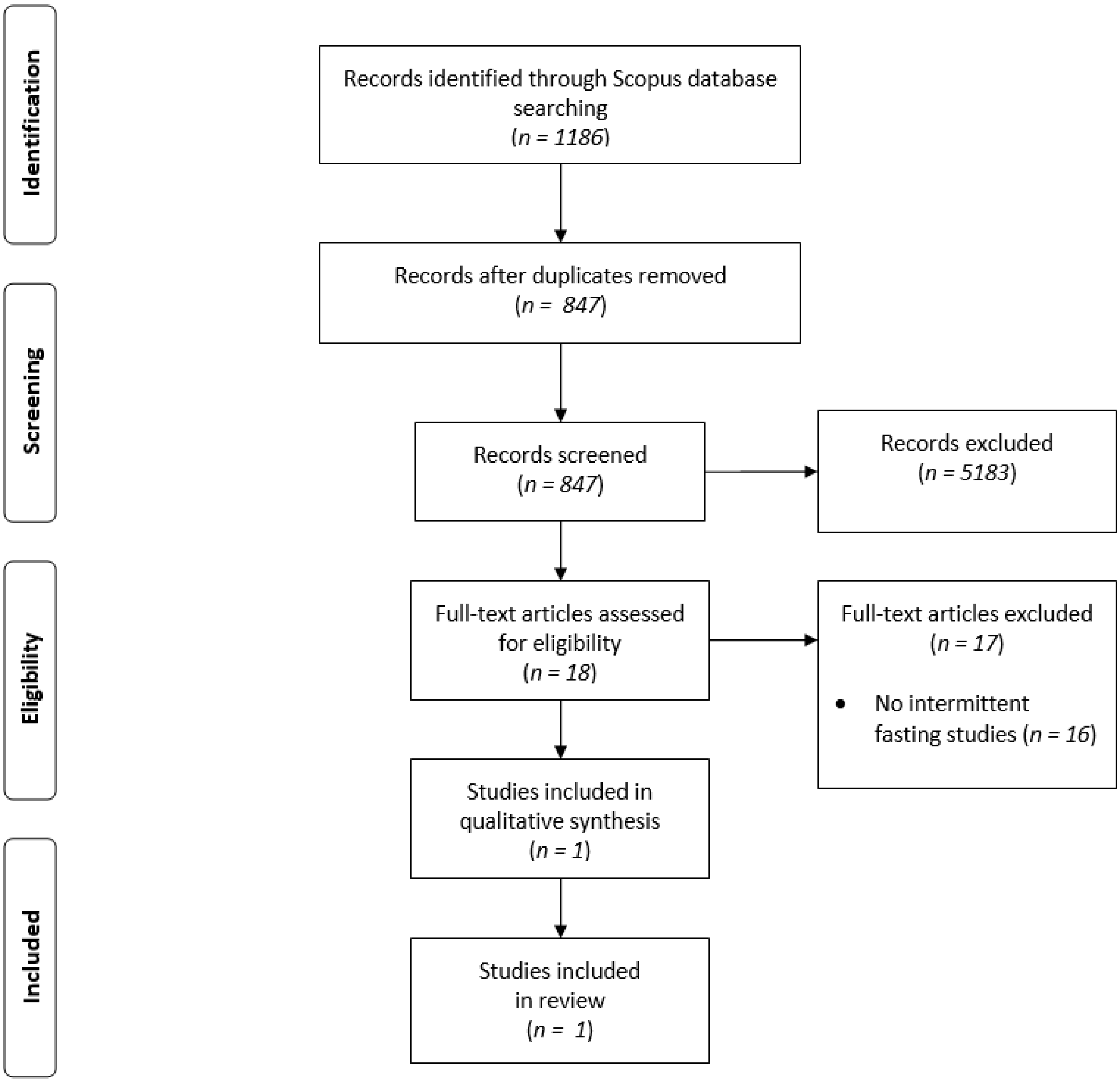

Figure S2. PRISMA flow diagram of the study selection from Scopus database.

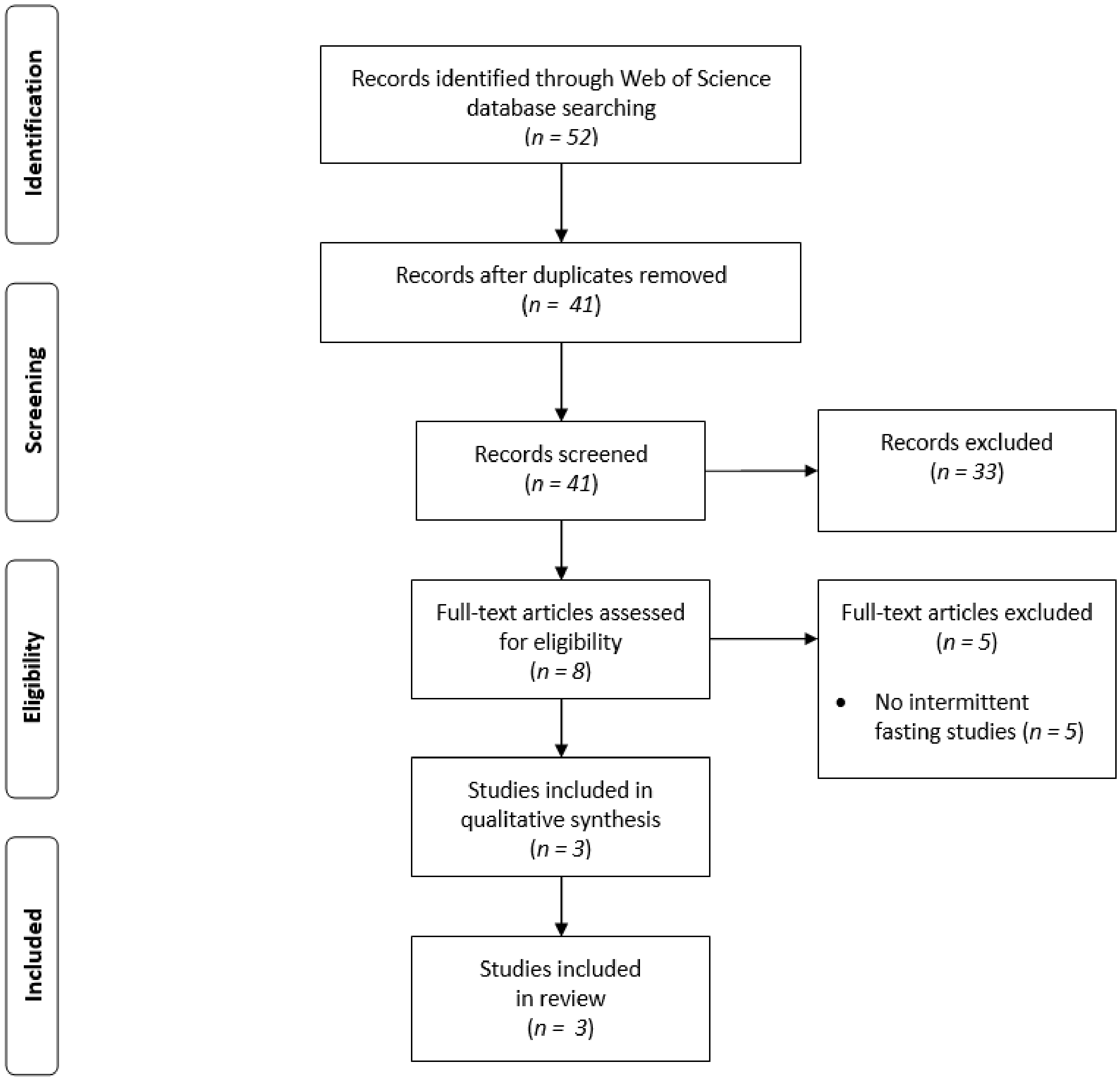

Figure S3. PRISMA flow diagram of the study selection from Web of Science database.
